# The impact of medications on salivary flow and oral health-related quality of life in postradiation head and neck cancer patients: results of the OraRad study

**DOI:** 10.1016/j.oooo.2025.06.019

**Published:** 2025-07-05

**Authors:** Adam M. Rose, Erika S. Helgeson, Kimberly C. Valentino, Rajesh V. Lalla, Nathaniel S. Treister, Brian L. Schmidt, Lauren L. Patton, Alexander Lin, Michael T. Brennan, Thomas P. Sollecito

**Affiliations:** a Department of Oral Medicine, University of Pennsylvania School of Dental Medicine, Philadelphia, PA, USA.; b Division of Biostatistics and Health Data Science, University of Minnesota, Minneapolis, MN, USA.; c Section of Oral Medicine, University of Connecticut Health, Farmington, CT, USA.; d Division of Oral Medicine and Dentistry, Brigham and Women’s Hospital, Boston, MA, USA.; e Department of Oral Medicine, Infection and Immunity, Harvard School of Dental Medicine, Boston, MA, USA.; f Department of Oral and Maxillofacial Surgery, New York University School of Dentistry, New York, NY, USA.; g Division of Craniofacial and Surgical Care, University of North Carolina, Adams School of Dentistry, Chapel Hill, NC, USA.; h Department of Radiation Oncology, University of Pennsylvania Perelman School of Medicine, Philadelphia, PA, USA.; i Department Oral Medicine/Oral & Maxillofacial Surgery, Atrium Health Carolinas Medical Center, Charlotte, NC USA.; j Division of Oral Medicine, Penn Medicine, Philadelphia, PA, USA.

## Abstract

**Objectives.:**

To determine the relationships between the number and class of xerogenic medications on whole stimulated salivary flow rates and oral health-related quality of life (OH-QOL) measures in patients who received high-dose external beam radiation therapy (RT) for head and neck cancer (HNC).

**Study Design.:**

Complete medication lists were generated using patient electronic health records from every attended study visit for 146 HNC patients. Whole stimulated salivary flow was measured before RT, and 6 and 18-months after RT. Ten single-item questions and two composite scales of swallowing problems and senses problems (taste and smell) were assessed at baseline and at 6-month intervals up to 24 months after RT. Linear mixed-effects models examined associations between the total number and class of medications and stimulated salivary flow and OH-QOL.

**Results.:**

There was no detected association between the total number of medications and stimulated salivary flow (p-value = .18). Only antidepressant usage was significantly associated with stimulated salivary flow (*P* = .006). Number of medications, narcotic analgesic, and antidepressant usage were significantly associated with a clinically meaningful decrease in OH-QOL.

**Conclusion.:**

Antidepressants were associated with reduced stimulated salivary flow, but no cumulative negative effect on whole stimulated salivary flow was identified. Polypharmacy was associated with worse OH-QOL.

Saliva plays an essential role in oral health by providing critical functions for digestion and in maintaining oral homeostasis, but disruption of salivary flow can be detrimental to an individual’s health and well-being.^1^ External beam radiation therapy (RT) is an effective treatment for head and neck cancer (HNC), but it can also produce significant functional and sensory changes in oral soft tissues like the salivary glands^2^ that often results in hyposalivation (decreased saliva production) and related morbidities, such as xerostomia (the subjective feeling of dry mouth) that negatively impact patient quality of life (QOL).^3^ Previous studies have demonstrated that RT decreases salivary flow and negatively impacts oral health-related QOL (OH-QOL), but also that partial recovery of salivary output is possible with time.^3,4,5^ However, numerous medications across commonly prescribed drug classes are also known to induce hyposalivation in patients^1,6^ and compromise the crucial protective and digestive functions of saliva.^7^ Therefore, xerogenic medications can produce the same adverse effects associated with radiation even without damage to the salivary glands,^8^ including decreased OH-QOL measures related to xerostomia.^9^

To date, the effect of medication usage on salivary output remains unclear in HNC patients who have undergone RT, and therefore should be studied to better guide care and improve the health outcomes of these patients. The Clinical Registry of Dental Outcomes in Head and Neck Cancer Patients (OraRad) study represents the largest prospective, longitudinal, multicenter cohort study of salivary flow and OH-QOL in HNC patients receiving high-dose RT with curative intent. The objective of this analysis from the OraRad study was to describe medication usage and its impact on salivary flow at 6 months and 18 months after RT, as well as associated patient-reported OH-QOL outcomes at 6-month intervals over the first two years after RT, for the patient cohort treated at one of the OraRad clinical centers.

## MATERIALS AND METHODS

### Study cohort

The OraRad study, previously described,^10^ enrolled HNC patients at six clinical centers: Brigham and Women’s Hospital, Atrium Health Carolinas Medical Center, New York University, University of Connecticut Health, University of North Carolina, and University of Pennsylvania. Institutional Review Board approval was obtained at all sites and participants were consented and enrolled before initiating curative-intent (definitive or postoperative) head and neck RT following the guidelines of the Helsinki Declaration. Patients were eligible if: age 18 or older; diagnosed with head and neck squamous cell carcinoma (SCC) or a salivary gland cancer (SGC), or with a non-SCC, non-SGC malignancy of the head and neck region; planned to receive at least 4500 cGy RT to the head and neck region; and had no prior RT to the head and neck region. A total of 572 participants were enrolled between April 2014 and October 2018 and eligible for follow up post-RT. The initial (baseline) study assessments occurred after a pre-radiation dental evaluation and any required extractions and before the first RT session. 146 HNC patients treated at the University of Pennsylvania from April 2014 to June 2019, representing a subset of the patients enrolled in the OraRad study, were included for the analysis detailed in this report.

### Study assessments

Whole stimulated salivary flow rate was measured at the baseline, 6-month, and 18-month study visits to assess the effect of RT on salivary flow. Participants were provided with unflavored paraffin wax (gum base) and two 50 ml test tubes. They were instructed to chew the gum base for 2 min, meanwhile expectorating saliva into one of two test tubes. This was done as practice to standardize the technique and stabilize the flow rate. The same chewing/expectorating method was used for 5 min for the final flow rate assessment, timed using a digital timer. The saliva collected in 5 min in the second test tube was weighed and recorded.

Patient-reported symptoms pertaining to OH-QOL were assessed at each study visit: at baseline prior to RT, and then 6-months, 12-months, 18-months, and 24-months post-RT, using selected single item questions and composite scales from the European Organization for Research and Treatment of Cancer Quality of Life Question (EORTC QLQ)-HNC specific module-35 items (H&N35) scale.^11^ Ten questions were scored on a four-point scale (1 = not at all; 2 = a little; 3 = quite a bit; 4 = very much) to evaluate a patient’s experience with their oral health the week before their study visit: problems swallowing liquids, pureed food, solid foods, choking when swallowing, problems with teeth, opening mouth, dry mouth, sticky saliva, problems with sense of smell, and sense of taste. Two composite scales were assessed: problems with swallowing (which summarizes the problems swallowing liquids, pureed food, and solid foods and choking when swallowing items) and senses problems (which summarizes the problems with sense of smell and taste items). Prior to analysis, the ten four-point scale items and two composite scales were transformed into 1-to-100 scales, with higher scores representing higher level of symptoms. These scales were treated as continuous in all analyses. A ≥10 unit difference on this 100 unit scale has been rated as clinically meaningful, as previously suggested.^11,12^

Complete medication lists were generated for each patient using electronic health records from every attended visit and reconciled by cross-examining these lists with recorded prescription start and end dates. Medications that were stopped on the day of a study visit were included with that visit, and medications that were prescribed on the day of a study visit were excluded. Dermatologic and ophthalmic topical medications, vitamins and other nutritional supplements, single-dose chemotherapeutics, and radiologic contrast agents were also excluded. Each individual patient’s completed medication list was then aggregated to determine which classes of medication were most prescribed among the patient cohort. The threshold for inclusion of a xerogenic drug class, based on results from the Xeromeds Consortium,^1^ was set to be 40 patients (i.e. medication class used by >27% of cohort) and yielded 11 classes of medications for consideration: non-narcotic analgesics (e.g., aspirin, ibuprofen, acetaminophen) (63.3%), narcotic analgesics (e.g., oxycodone, hydromorphone) (55.1%), anticonvulsants (e.g., gabapentin, pregabalin) (50.3%), proton pump inhibitors/H2-blockers (e.g., omeprazole, esomeprazole) (42.3%), antihypertensives (e.g., lisinopril, metoprolol) (42.2%), antilipids (e.g., atorvastatin, rosuvastatin) (40.1%), corticosteroids (e.g., dexamethasone, prednisone) (31.3%), benzodiazepines (e.g., lorazepam, alprazolam) (30.6%), non-sex hormones (e.g., levothyroxine, melatonin) (29.9%), laxatives (e.g., docusate sodium, polyethylene glycol) (27.9%), and anti-depressants (e.g., escitalopram, sertraline) (27.2%).

### Statistical considerations

Linear mixed-effects models with subject specific random intercepts were used to evaluate the relationship between medication measures and salivary flow across relevant study time points (baseline, 6-months, and 18-months) and QOL measures across all study time points (baseline, 6-months, 12-months, 18-months, and 24-months). The interaction between the medication measure and study visits (treated as categorical) was first assessed to evaluate whether the relationship between medication usage and outcome depended on the timing relative to RT. If the interaction was not significant (at a *P* = .01 threshold), it was removed from the model with the final model including main effects for study visits and the medication measure. Analyses evaluating the association between medication class usage and outcomes did not account for the number of medications in a given class or the other medications that the patient might be taking.

Trends were visualized using locally estimated scatterplot smoothing (loess) lines.^13^ Analyses were conducted using R version 4.3.1^14^ using versions 1.1.34, 3.1.3, and 1.8.8 of the “lme4,”^15^ “lmerTest,”^16^ and “emmeans”^17^ packages, respectively. All p-values are two-sided and have not been adjusted for multiple comparisons. Given the many analyses presented, a p-value threshold of 0.01 was used for considering a result statistically significant.

## RESULTS

### Patient demographic and medication characteristics

A total of 146 patients were included, with a median (interquartile range) age at baseline visit of 58 (51, 63) years, and the majority being male and White (81.5% and 91.8%, respectively) (Table 1). Squamous cell carcinoma was the most common pathology (82.2%), and oropharynx the most common primary site for RT (49.3%). While the majority of patients (64.4%) presented with early tumor stage (T1/T2) disease, regional lymph node involvement was common (76.7%) (Table 1). The median (interquartile range) total RT dose delivered to the primary tumor site was 6300 (6000, 7000) cGy delivered over 30 (30, 35) fractions. Most RT fields included the nodal regions of the neck (93.2%) with bilateral neck RT treatment in 85.3% of cases. Additionally, 50.7% of patients received chemotherapy and 77.4% received surgery prior to RT.

At baseline, the median (quartiles) number of medications was 3.0 (2.0, 5.0), with the median number of medications remaining constant at the population level across study visits (Table 2). At baseline, the most commonly prescribed medications were non-narcotic analgesics (47.3% usage) and the least commonly prescribed were hormones (8.2% usage). At 6-months, the most commonly prescribed medications were antihypertensives (38.2% usage) and the least commonly prescribed were hormones (10.7%). At 12-months, the most commonly prescribed medications were non-narcotic analgesics (42.7%) and the least commonly prescribed were laxatives (14.5%). At 18-months, the most commonly prescribed medications were non-narcotic analgesics (44.3%) and the least commonly prescribed were laxatives (13.4%). At 24-months, the most commonly prescribed medications were non-narcotic analgesics (42.1%) and the least commonly prescribed were laxatives (10.5%).

### Medication usage, salivary flow, and EORTC QOL measures

From baseline to 6-months, 24.5% of individuals experienced no change in the number of medications, 39.7% decreased the number of medications, and 35.9% increased the number of medications (Table 2). Results were similar for changes from baseline to 12-, 18- and 24-months.

**Fig. 1.**
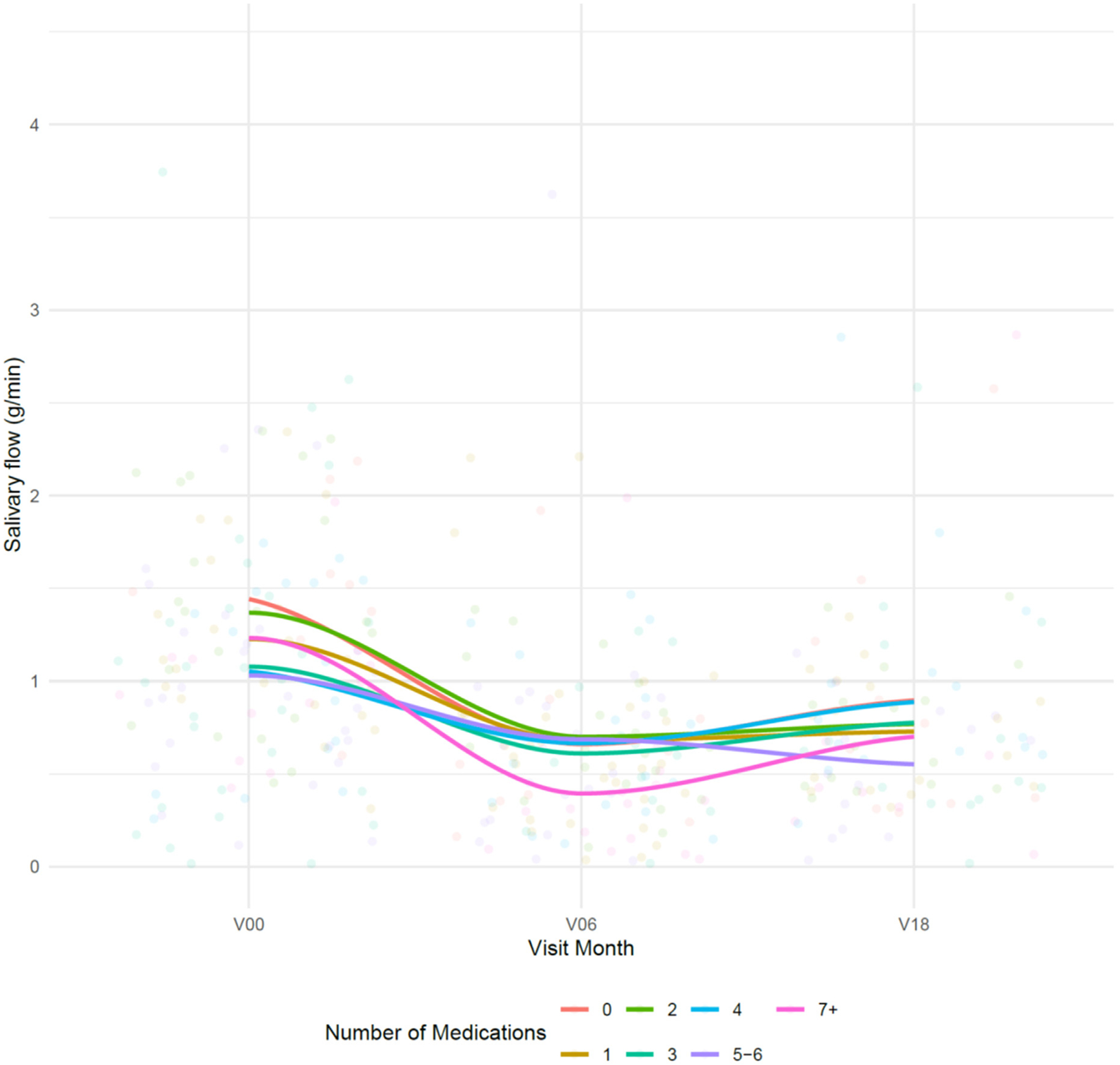
Loess curves illustrating relationship between number of medications and salivary flow. Visit month has been jittered for visualization purposes. Statistical comparisons are provided in Table 3.

No significant association between the total number of medications and salivary flow was detected (*P* = .66 for changes in association relative to RT, *P* = .18 for association across study visits; Figure 1, Supplemental Table S1, Table 3). Furthermore, we did not identify that the association between medication usage and salivary flow depended on the timing of usage relative to RT (p-values > .10, Supplementary Table S1). Of the 11 medication classes considered, antidepressant usage was associated with salivary flow, with usage associated with a 0.20 (95% CI: 0.06–0.35) g/min reduction in salivary flow.

**Fig. 2.**
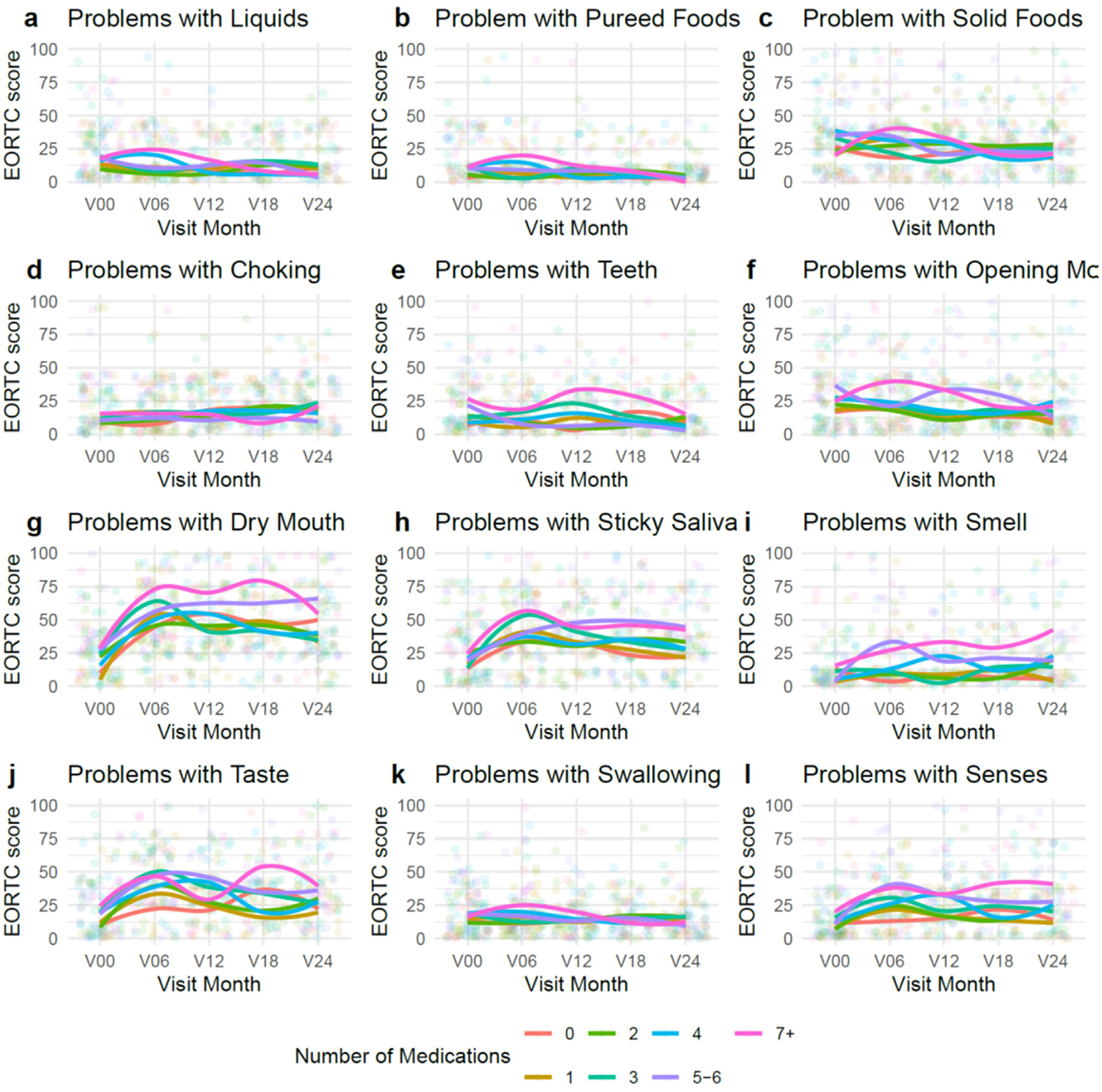
Loess curves illustrating the relationship between number of medications and OH-QOL measures. Visit month has been jittered for visualization purposes. Statistical comparisons provided in Table 3.

We did not identify that the association between number of medications and QOL measures depended on the timing of usage relative to RT (p-values > .01, Supplementary Table S1). At a given visit, number of medications was associated with increased problems swallowing liquids (*P* = .002), increased problems swallowing pureed foods (*P* = .002), increased problems with teeth (*P* = .001), increased difficulty opening mouth (*P* = .002), increased sticky saliva (*P* = .009), increased problems with sense of smell (*P* < .001), increased problems with sense of taste (*P* = .007), increased problems swallowing (*P* = .009), and increased senses problems (*P* < .001; Table 3, Figure 2). Problems with smell had the strongest association with number of medications, with 1 additional medication associated with an increase of 1.7 (95% CI: 0.8, 2.6) on the problems with smell scale. This equates to approximately a difference of 6 medications being associated with a clinically meaningful (10 point) difference in problems with sense of smell.

We identified that the association between narcotic analgesics and difficulty swallowing pureed food depended on the timing relative to RT (*P* = .009). Specifically, individuals taking narcotic analgesics at the 6-month visit experienced a 16.6 (95% CI: 9.8, 23.4) unit greater difficulty swallowing pureed food compared to individuals not taking that class of medications at the 6-month visit (Supplemental Table S2; Supplemental Figure S1). Difficulty swallowing pureed foods was not associated with taking narcotic analgesics at baseline, 12-months, 18-months, or 24-months.

Antidepressants were associated with a 12.0 (5.6, 18.5) unit greater difficulty swallowing solid foods. While not clinically significant (i.e., ≥10 unit difference), antidepressants were also associated with a 7.0 (95% CI: 2.2, 11.8) unit greater difficulty swallowing liquids, 6.4 (95% CI: 2.3, 10.5) unit greater difficulty swallowing pureed foods, and a 7.4 (95% CI: 3.4, 11.4) unit higher swallowing scale score. Additional relationships between medication usage and various EORTC QOL measures were identified but did not meet the threshold for clinical significance (Table 3).

## DISCUSSION

We investigated the relationships between the number and class of xerogenic medications on whole stimulated salivary flow rates and on OH-QOL in patients who received high-dose external RT for HNC. This cohort study describes the variety of medications taken by HNC patients and shows that these medications are associated with a negative impact on OH-QOL in the months immediately following RT. Patients taking antidepressants had lower salivary flow rates than patients not taking these medications, but all other analyses evaluating the total number of medications and class of medications and salivary flow were not statistically significant. However, the number of medications was associated with many aspects of OH-QOL including problems with swallowing, senses problems, problems with teeth, problems opening mouth wide, and sticky saliva. Evaluating the specific classes of medications, we identified that narcotic analgesics were associated with a clinically meaningful greater difficulty swallowing pureed food, but only at the 6-month visit, and antidepressant usage was associated with a clinically meaningful greater increase in problems swallowing solid foods across study visits.

Prior research has demonstrated that various classes of medication can disrupt salivary gland function, and that taking multiple xerogenic medications is linked to decreased flow rates of both unstimulated and stimulated saliva.^1,7,18,19^ However, none of the patients in these previous investigations had a history of HNC, and patients with radiation-induced xerostomia were excluded in three of the study designs.^1,18,19^ To our knowledge, the effect of xerogenic medication intake on salivary gland function in the context of HNC treatment has not been previously investigated. Therefore, the negative results of our analyses evaluating the total number of medications and whole stimulated salivary flow that contradict previous findings likely reflect these fundamental differences in the patient populations from our cohort compared to those in other studies. It is important to note that this cohort has deficient salivary production and decreased functional reserve capacity from RT compared to patients without HNC, likely leaving less glandular tissue that is susceptible to further impact from xerogenic meds. Another explanation may be that stimulated salivary flow can negate the anticholinergic effects of these medications, and therefore we did not observe any associations in our study because we only measured whole stimulated salivary flow and did not consider unstimulated salivary flow.

The significant reduction to salivary flow by antidepressant use supports prior findings.^1^ A systematic review by Wolff et al.^6^ identified 56 medications with strong evidence of affecting salivary gland function, including 14 antidepressants, the most of any drug class for this highest level of evidence; another 50 medications with moderate evidence of affecting salivary gland function included an additional 4 antidepressants. However, these same studies also reported that corticosteroid use was associated with reduced whole stimulated salivary flow, a finding that was not observed in this study (*P* = .20).

Multiple studies have documented both short and long-term negative impacts to OH-QOL from various HNC radiation techniques using patient-reported outcomes, including changes in salivary flow and swallowing challenges.^20,21,22,23,24^ We previously reported that OH-QOL, as assessed by the ten single-item questions and two composite scales, demonstrated variable levels of change over the 2-year study period, with senses problems (including problems with taste), sticky saliva, and dry mouth having clinically meaningful (≥ 10 unit) changes.^20^ This is consistent with the finding by Likhterov et al.^22^ that among patients receiving RT, changes in EORTC scores for dry mouth, sticky saliva and senses accompanied initial decline to saliva and later improved after 36 months. Similarly, it is well-established that xerogenic medications and the corresponding hyposalivation negatively impact QOL^25,26^ in both young and geriatric patient cohorts.^27,28^

There are several limitations in our study that are important to mention. Given the modest sample size and considerable number of classes of drugs evaluated, we were unable to account for other drug class usage in our analyses evaluating the association between a specific drug class and outcomes. Another limitation is that patient medication lists relied on reviewing prescription documentation in electronic health records. Though the lists were reconciled, patients may not have filled their prescriptions and may not have been taking the medications, or may have been taking over-the-counter medications that were not documented in their patient chart and therefore unknown to the study. The number of medications individuals took varied from visit to visit, and thus it was impossible to create matched patient groups for the analyses evaluating all of the medications. Hence, some true associations may be masked and some identified associations may be due to confounding. Also, our analyses were restricted to evaluating usage vs non-usage for medication classes compared to evaluating whether the number of medications in a given class was associated with outcomes. Patient follow-up was limited to up to 2 years, with only two post-RT timepoints for measuring salivary flow (6-months and 18 months). Improvements in salivary flow and patient-reported outcomes, observed with time, suggest that recovery may be possible for patients during post-treatment follow-up.^3,29^ Furthermore, it is possible that clinical changes to the oral cavity and their subsequent impact on OH-QOL is a gradual process that will be more impactful for patient QOL in later years of survivorship. Thus, to better understand the long-term effects of RT, current efforts are underway to complete a longer follow-up of up to 7 years for the OraRad study cohort. However, the results will still be imperfect because QOL measures are subject to recall bias by participants. Additionally, while we set a stricter threshold for statistical significance than is typical (p-value < .01 vs .05), we must caution that the analyses are exploratory in nature and prone to increased type-I error given the multiple analyses conducted in this paper.

The patient cohort presented in this publication is a portion (25.5%) of the OraRad study cohort, specifically those treated and followed up at the University of Pennsylvania. Overall, most of the baseline characteristics seen in this University of Pennsylvania group (Table 1) are representative of the full OraRad cohort.^30^ The notable differences are a greater percentage of males represented (81.5% vs 76.9%), percentage of patients who received chemotherapy (63.6% vs 50.7%), and percentage of patients who received surgery (77.4 vs 54.9%). The sex distribution is relevant as a potential confounder because we have demonstrated for the full OraRad cohort that females with HNC had significantly lower whole stimulated salivary flow than males at each study visit, both before and after RT.^31^ However, because there were only 27 females (18.5%) compared to 119 males (81.5%) in the current analysis, there was inadequate statistical power for separate comparisons by sex of the interactions between salivary flow and the number/type of medications.

## CONCLUSIONS

This investigation is the first to consider the role of xerogenic medications in modulating salivary flow and impacting HNC patient QOL within the context of treatment with high-dose RT. These analyses found that antidepressants were associated with reduced stimulated salivary flow more than other drug classes within this patient cohort, but there was no cumulative negative effect on whole stimulated salivary flow for HNC patients taking multiple xerogenic medications. Polypharmacy was associated with lower OH-QOL. The results of these analyses and further studies may help inform medication prescription practices to improve HNC patient quality of life during recovery from high-dose RT.

## Supplementary Material

Supplement 1A

Supplement 1B

Supplement 2

Supplement 3

Supplementary material associated with this article can be found in the online version at doi:10.1016/j.oooo.2025.06.019.

## Figures and Tables

**Table 1.** Demographics and clinical characteristics of the study population

	Overall (N = 146)
Age (median [IQR])	58 [51, 63]
Sex (%)	
Female	27 (18.5)
Male	119 (81.5)
Race (%)	
Black Only	6 (4.1)
Other	6 (4.1)
White Only	134 (91.8)
Ethnicity (%)	
Hispanic	1 (0.7)
Not Hispanic	145 (99.3)
Education (%)	
At most high school	42 (28.8)
Greater than high school	104 (71.2)
Marital status (%)	
Married	114 (78.1)
Not Married	32 (21.9)
Public Assistance (%)	
No	138 (94.5)
Yes	8 (5.5)
Insurance (%)	
Medicaid only	2 (1.4)
Medicare/Medicare Part B and Private	24 (16.4)
Medicare/Medicare part B only	3 (2.1)
No insurance	1 (0.7)
Private only	114 (78.1)
Unknown	2 (1.4)
Dental Insurance (%)	
No	36 (24.7)
Yes	110 (75.3)
Tobacco use (%)	
Ever Used	82 (56.2)
Never used	64 (43.8)
Alcohol use (%)	
Alcohol use in past 12 months	106 (72.6)
No alcohol use in past 12 months	40 (27.4)
Drinks per week (median [IQR])	2 [0, 6]
Histology (%)	
non-SCC, non-SGC	7 (4.8)
SCC	120 (82.2)
SGC	19 (13.0)
Primary site of RT (%)	
Larynx/Hypopharynx	6 (4.1)
Oral Cavity	12 (8.2)
Oropharynx	72 (49.3)
Other	38 (26.0)
Salivary gland	18 (12.3)
Tumor stage (%)	
1 or 2	94 (64.4)
3or4	46 (31.5)
Other	6 (4.1)
Distant metastases (%)	
Absent	146 (100.0)
Nodal involvement (%)	
00	33 (22.6)
01/02/2a/2b/2c/03	112 (76.7)
Other	1 (0.7)
Total number of radiation fractions delivered (median [IQR])	30 [30, 35]
Dose per fraction (cGy) (median [IQR])	200 [200, 200]
Total dose to primary site (cGy) (median [IQR])	6300 [6000, 7000]
RT treatment to neck (%)	
Unknown	1 (0.7)
Did not receive next RT	9 (6.2)
Received neck RT	136 (93.2)
Bilateral	116 (85.3)
Unilateral	20 (14.7)
Chemotherapy (%)	
No	72 (49.3)
Yes	74 (50.7)
Surgery pre-RT (%)	
No	33 (22.6)
Yes	113 (77.4)

IQR, interquartile ranges; SCC, squamous cell carcinoma; SGC, salivary gland cancer; RT, Radiation therapy; cGY, centigray.

**Table 2.** Medication usage across study visits.

	Baseline (N = 146)	6-months (N = 131)	12-months (N = 110)	18-months (N = 97)	24-months (N = 114)
Total number of medications (median [IQR])	3.0 [2.0, 5.0]	3.0 [1.0, 5.0]	3.0 [1.0, 4.0]	3.0 [1.0, 4.0]	3.0 [1.0, 4.0]
Change in medications from BL (%)					
Decrease		52 (39.7)	38 (34.5)	35 (36.1)	43 (37.7)
No Change		32 (24.4)	32 (29.1)	28 (28.9)	33 (28.9)
Increase		47 (35.9)	40 (36.4)	34 (35.1)	38 (33.3)
Medication Usage					
Non-narcotic analgesic (%)	69 (47.3)	47 (35.9)	47 (42.7)	43 (44.3)	48 (42.1)
Narcotic analgesic (%)	66 (45.2)	27 (20.6)	18 (16.4)	13 (13.4)	13 (11.4)
Anticonvulsant (%)	25 (17.1)	49 (37.4)	28 (25.5)	20 (20.6)	19 (16.7)
Antihypertensive (%)	60 (41.1)	50 (38.2)	38 (34.5)	33 (34.0)	40 (35.1)
Antilipid (%)	42 (28.8)	39 (29.8)	34 (30.9)	40 (41.2)	41 (36.0)
Corticosteroids (%)	23 (15.8)	26 (19.8)	22 (20.0)	19 (19.6)	23 (20.2)
PPI/H2 (%)	40 (27.4)	38 (29.0)	31 (28.2)	32 (33.0)	33 (28.9)
Benzodiazepines (%)	31 (21.2)	28 (21.4)	19 (17.3)	16 (16.5)	15 (13.2)
Hormones (%)	12 (8.2)	14 (10.7)	26 (23.6)	18 (18.6)	35 (30.7)
Laxatives (%)	24 (16.4)	18 (13.7)	16 (14.5)	13 (13.4)	12 (10.5)
Antidepressants (%)	22 (15.1)	33 (25.2)	25 (22.7)	22 (22.7)	21 (18.4)

IQR, interquartile range; BL, baseline; PPI/H2, proton pump inhibitors and H2 receptor blockers.

**Table 3.** Association between medication usage and EORTC QOL measures and salivary flow.

	Problems Swallowing Liquids	Problems Swallowing Pureed Foods	Problems Swallowing Solid Foods	Choked when swallowing	Problems with Teeth	Problems Opening Mouth Wide	Dry Mouth	Sticky Saliva	Problems with Sense of Smell	Problems with Sense of Taste	Problems Swallowing	Senses problems	Salivary flow (g/min)
Total number of meds	**1.3 (0.5, 2.0); 0.0017**	**1.1 (0.4, 1.8); 0.0015**	0.7 (−0.3, 1.7); 0.1853	−0.1 (−0.9, 0.6); 0.7009	**1.2 (0.5, 2); 0.0014**	**1.5 (0.6, 2.5); 0.0024**	1.4 (0.3, 2.5); 0.0141	**1.4 (0.4, 2.5); 0.0088**	**1.7 (0.8, 2.6); 2e-04**	**1.5 (0.4, 2.5); 0.007**	**0.9 (0.2, 1.5); 0.009**	**1.6 (0.8, 2.4); 2e-04**	−0.02 (−0.04, 0.01); 0.1828
Non-narcotic Analgesic	3.2 (−0.5, 6.8); 0.0906	0.9 (−2.3, 4.1); 0.581	−1.8 (−6.6, 2.9); 0.4473	−1.9 (−5.3, 1.6); 0.2835	1.5 (−2.2, 5.2); 0.4397	4.5 (0, 9); 0.0519	1.3 (−3.9, 6.5); 0.6257	3.8 (−1.4, 8.9); 0.1519	−1.8 (−5.9, 2.4); 0.4048	4 (−1, 9); 0.1221	0.5 (−2.5, 3.4); 0.7637	1.3 (−2.6, 5.2); 0.5053	−0.02 (−0.13, 0.09); 0.766
Narcotic Analgesic	**7.5 (3.5, 11.4); 2e-04**	*	**7 (2, 12.1); 0.0068**	1 (−2.7, 4.7); 0.6048	1.5 (−2.7, 5.8); 0.4708	5.8 (1, 10.7); 0.018	6.7 (1, 12.3); 0.0208	1.9 (−3.9, 7.6); 0.5289	3.5 (−0.9, 7.9); 0.1204	4 (−1.5, 9.6); 0.1569	**5.6 (2.5, 8.8); 5e-04**	3.6 (−0.6, 7.8); 0.0928	−0.06 (−0.17, 0.05); 0.2834
Anti-convulsant	2.8 (−1.2, 6.8); 0.1728	−0.4 (−4, 3.2); 0.8159	2.5 (−2.6, 7.7); 0.3382	−1.9 (−5.7, 1.8); 0.3111	1.1 (−3.1, 5.3); 0.5973	4.3 (−0.7, 9.2); 0.0895	6.3 (0.5, 12); 0.0321	1.8 (−3.9, 7.6); 0.5354	3.3 (−1.2, 7.7); 0.1558	1.6 (−4, 7.1); 0.5817	0.9 (−2.3, 4.2); 0.5733	2.5 (−1.8, 6.8); 0.2551	−0.05 (−0.16, 0.07); 0.4155
Anti-hypertensive	−2 (−6.6, 2.5); 0.385	−1.2 (−5, 2.5); 0.5168	−5.3 (−11.5, 1); 0.0986	−2.6 (−7, 1.9); 0.2602	1.3 (−3.2, 5.6); 0.5742	6.2 (0, 12.5); 0.048	−1.6 (−8.6, 5.2); 0.6372	4 (−2.2, 10.2); 0.2048	−1.2 (−6.8, 4.3); 0.6623	2 (−4.4, 8.3); 0.5284	−2.5 (−6.5, 1.4); 0.2131	0 (−5.1, 5.1); 0.9878	0.11 (−0.03, 0.25); 0.1328
Antilipid	−4 (−8.4, 0.3); 0.0672	−1.5 (−5.1, 2.2); 0.4323	−7.4 (−13.2, −1.7); 0.012	−3.5 (−7.6, 0.7); 0.1002	0.1 (−4.2, 4.4); 0.9601	−1 (−6.6, 4.6); 0.7294	3.5 (−2.8, 9.8); 0.2839	2.1 (−4, 8.1); 0.5054	6 (0.9, 11.1); 0.0207	6 (0, 11.9); 0.0497	−4 (−7.6, −0.3); 0.0333	6 (1.3, 10.7); 0.0128	0.06 (−0.07, 0.19); 0.3876
Corticosteroids	2.6 (−1.9, 7.1); 0.2555	1.5 (−2.5, 5.5); 0.4687	2.1 (−3.7, 7.9); 0.474	−2.7 (−6.8, 1.5); 0.2158	−0.9 (−5.6, 3.7); 0.6893	1.2 (−4.3, 6.7); 0.6718	5.3 (−1, 11.7); 0.1019	−3.4 (−9.7, 3); 0.2974	3.4 (−1.7, 8.4); 0.1917	−3.5 (−9.7, 2.7); 0.2746	1.5 (−2.1, 5.2); 0.4124	0 (−4.8, 4.9); 0.9845	−0.09 (−0.23, 0.05); 0.1965
PPI/H2	0.7 (−3.4, 4.8); 0.7391	1.4 (−2.1, 4.9); 0.4307	5.7 (0.4, 11); 0.0356	−1.3 (−5.2, 2.5); 0.5024	2.3 (−1.8, 6.4); 0.2743	3.2 (−1.9, 8.2); 0.2265	4.3 (−1.6, 10.1); 0.1532	6.7 (1, 12.4); 0.0228	5.1 (0.4, 9.8); 0.0303	**7.8 (2.2, 13.4); 0.0065**	1.3 (−2, 4.7); 0.4334	**6.4 (2, 10.8); 0.0038**	−0.07 (−0.19, 0.04); 0.2133
Benzo-diazepines	3.2 (−1.6, 8); 0.1924	**6.1 (2, 10.1); 0.004**	−0.7 (−7.1, 5.6); 0.8161	−2.1 (−6.7, 2.5); 0.3725	1.1 (−3.8, 5.9); 0.6625	2.7 (−3.4, 8.8); 0.3826	−1 (−8.1, 6); 0.7677	0.6 (−6.2, 7.4); 0.8583	5.1 (−0.4, 10.6); 0.0695	4.3 (−2.4, 10.9); 0.2103	1.5 (−2.4, 5.5); 0.4492	4.5 (−0.7, 9.6); 0.0928	−0.01 (−0.16, 0.14); 0.8718
Hormones	0.6 (−4.2, 5.3); 0.8113	1.6 (−2.6, 5.8); 0.4632	0.4 (−5.7, 6.6); 0.887	1.4 (−3.1, 5.8); 0.5483	3.2 (−1.6, 8.1); 0.1956	−2.2 (−8.1, 3.6); 0.4559	−0.5 (−7.3, 6.3); 0.8906	5.8 (−1, 12.6); 0.092	5.9 (0.6, 11.2); 0.0314	5.2 (−1.4, 11.7); 0.1246	1.1 (−2.7, 5); 0.5634	5.6 (0.6, 10.7); 0.03	0.1 (−0.07, 0.26); 0.2454
Laxatives	6.2 (1.4, 11); 0.0107	**7.2 (2.9, 11.4); 0.001**	7 (0.9, 13.2); 0.0254	3.7 (−0.8, 8.2); 0.105	5.3 (0.4, 10.3); 0.0355	6.7 (0.9, 12.6); 0.0242	1.4 (−5.4, 8.2); 0.6844	**9.1 (2.3, 16); 0.0095**	5.3 (0, 10.6); 0.0531	2.4 (−4.3, 9); 0.4886	**5.8 (2, 9.6); 0.0033**	3.7 (−1.4, 8.8); 0.1539	−0.07 (−0.22, 0.07); 0.303
Anti-depressants	**7 (2.2, 11.8); 0.0048**	**6.4 (2.3, 10.5); 0.0026**	**12 (5.6, 18.5); 3e-04**	2.9 (−1.8, 7.5); 0.2272	3.9 (−1, 8.7); 0.1142	3.6 (−2.7, 9.9); 0.2552	6.8 (−0.2, 13.8); 0.0578	6.1 (−0.6, 12.9); 0.0773	2.7 (−2.9, 8.4); 0.3432	−4.6 (−11.3, 2.1); 0.1795	**7.4 (3.4, 11.4); 4e-04**	−1 (−6.3, 4.2); 0.7003	−**0.2 (−0.35, −0.06); 0.0062**

* A significant interaction between study visit and medication use was identified. Results are presented in Supplemental Table S2 and Figure S1 Estimate (95% CI); p-value presented for association between one additional medication (total number of meds) or usage of class of medication and outcomes from a linear mixed effects model adjusting for study visit. Associations with p-value<0.01 in bold.

## Statement of Clinical Relevance

This study investigated how xerogenic medications impact salivary flow and quality of life in head and neck cancer patients treated with high-dose radiation therapy. Antidepressants were associated with reduced stimulated salivary flow and polypharmacy correlated with diminished quality of life.
