# Supplementary figures and images for: The impact of medications on salivary flow and oral health-related quality of life in postradiation head and neck cancer patients: results of the OraRad study
Source: Oral Surg Oral Med Oral Pathol Oral Radiol. Author manuscript; Available in PMC 2026 Apr 5. (PMC13050463; doi:10.1016/j.oooo.2025.06.019)

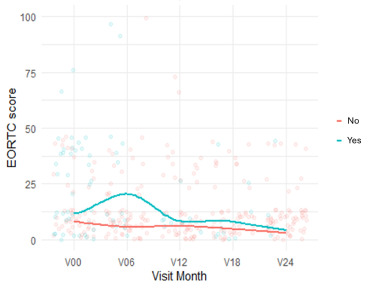

Supplement: Supplement 1A [file NIHMS2157814-supplement-Supplement_1A.jpg]

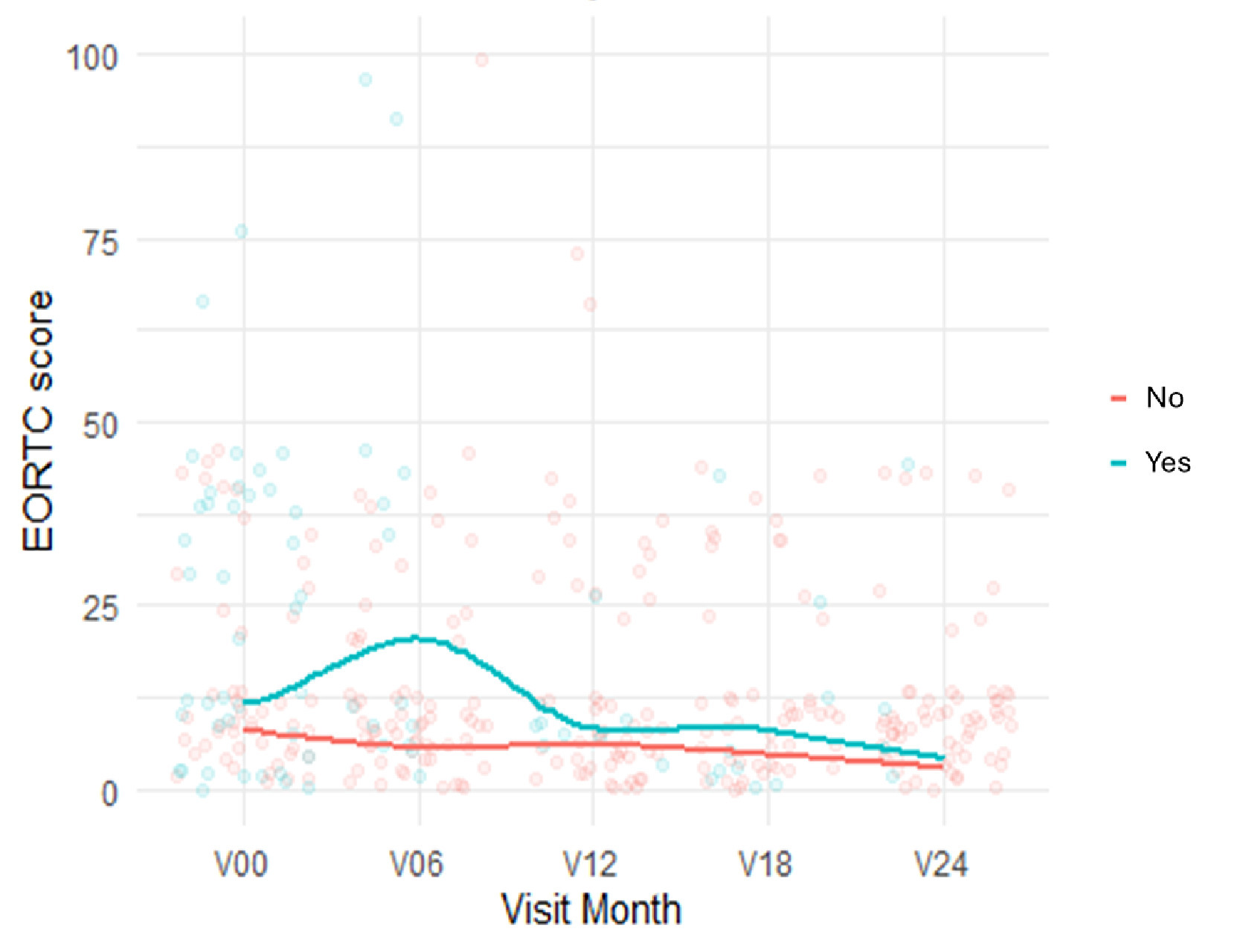

Supplement: Supplement 1B [file NIHMS2157814-supplement-Supplement_1B.jpg]
