# Supplementary material for: The impact of medications on salivary flow and oral health-related quality of life in postradiation head and neck cancer patients: results of the OraRad study
Source: Oral Surg Oral Med Oral Pathol Oral Radiol. Author manuscript; Available in PMC 2026 Apr 5. (PMC13050463; doi:10.1016/j.oooo.2025.06.019)
Supplement: Supplement 2 [file NIHMS2157814-supplement-Supplement_2.docx]

**Supplementary Table S1:** P-values for omnibus test of interaction between type of medication use and visit on EORTC values and salivary flow.

|  | Problems Swallowing Liquids | Problems Swallowing Pureed Foods | Problems Swallowing Solid Foods | Choked when swallowing | Problems with Teeth | Problems Opening Mouth Wide | Dry Mouth | Sticky Saliva | Problems with Sense of Smell | Problems with Sense of Taste | Problems Swallowing | Senses problems | Salivary flow (g/min) |
| --- | --- | --- | --- | --- | --- | --- | --- | --- | --- | --- | --- | --- | --- |
| Total number of meds | 0.0108 | 0.0226 | 0.4795 | 0.8687 | 0.0292 | 0.5007 | 0.9194 | 0.3451 | 0.0474 | 0.8495 | 0.0765 | 0.5702 | 0.6618 |
| Non-narcotic Analgesic | 0.0562 | 0.2322 | 0.6019 | 0.7048 | 0.5899 | 0.2691 | 0.4891 | 0.208 | 0.4061 | 0.7166 | 0.353 | 0.9275 | 0.4265 |
| Narcotic Analgesic | 0.0146 | **0.0084*** | 0.5807 | 0.9555 | 0.4102 | 0.2141 | 0.4551 | 0.074 | 0.1622 | 0.1184 | 0.0579 | 0.198 | 0.817 |
| Anti-convulsant | 0.1883 | 0.448 | 0.7254 | 0.4046 | 0.6223 | 0.2937 | 0.574 | 0.3431 | 0.5959 | 0.5016 | 0.8203 | 0.7633 | 0.9113 |
| Anti-hypertensive | 0.0772 | 0.5438 | 0.5259 | 0.831 | 0.0505 | 0.296 | 0.9255 | 0.2082 | 0.0148 | 0.1269 | 0.409 | 0.028 | 0.8474 |
| Antilipid | 0.7142 | 0.8974 | 0.8921 | 0.0525 | 0.2403 | 0.8311 | 0.5914 | 0.8982 | 0.5842 | 0.8014 | 0.9942 | 0.8749 | 0.8985 |
| Cortico-  steroids | 0.7361 | 0.4690 | 0.0651 | 0.2394 | 0.8685 | 0.0786 | 0.4504 | 0.1035 | 0.2697 | 0.2303 | 0.085 | 0.1241 | 0.1017 |
| PPI/H2 | 0.4102 | 0.1723 | 0.2055 | 0.6993 | 0.6301 | 0.3647 | 0.9495 | 0.1723 | 0.1074 | 0.5722 | 0.2491 | 0.3748 | 0.7037 |
| Benzo-diazepines | 0.2141 | 0.0739 | 0.0798 | 0.0758 | 0.1925 | 0.7366 | 0.5125 | 0.7175 | 0.1247 | 0.3567 | 0.0226 | 0.1973 | 0.9169 |
| Hormones | 0.7288 | 0.7734 | 0.5757 | 0.2028 | 0.953 | 0.8015 | 0.4563 | 0.1223 | 0.7868 | 0.9965 | 0.5928 | 0.9425 | 0.8838 |
| Laxatives | 0.3282 | 0.0291 | 0.6525 | 0.5809 | 0.436 | 0.0658 | 0.8617 | 0.3789 | 0.5452 | 0.9334 | 0.3667 | 0.8809 | 0.814 |
| Anti-depressants | 0.1071 | 0.3327 | 0.2395 | 0.3062 | 0.1954 | 0.7206 | 0.4866 | 0.0732 | 0.5606 | 0.8635 | 0.1471 | 0.6991 | 0.2356 |

*Described in Supplemental Table S2

Associations with p-value < 0.01 in bold. If the interaction was not significant (at a p-value = 0.01 threshold), it was removed from the model with the final model including main effects for study visits and the medication measure (see Table III)
