# Supplementary material for: The impact of medications on salivary flow and oral health-related quality of life in postradiation head and neck cancer patients: results of the OraRad study
Source: Oral Surg Oral Med Oral Pathol Oral Radiol. Author manuscript; Available in PMC 2026 Apr 5. (PMC13050463; doi:10.1016/j.oooo.2025.06.019)
Supplement: Supplement 3 [file NIHMS2157814-supplement-Supplement_3.docx]

**Supplemental Table S2:** Difference in problems with pureed foods at the specified study visit by narcotic analgesic usage. Estimate (95% CI) presented

| Visit | Usage | No usage | Difference |
| --- | --- | --- | --- |
| Baseline | 10.9 (6.9, 14.9) | 8.9 (5.3, 12.6) | 2 (-3.1, 7.2) |
| 6-months | 22.6 (16.4, 28.8) | 6 (2.7, 9.3) | 16.6 (9.8, 23.4) |
| 12-months | 12 (4.2, 19.8) | 6.1 (2.7, 9.6) | 5.9 (-2.5, 14.3) |
| 18-months | 13.7 (5.1, 22.3) | 5.4 (1.8, 9) | 8.3 (-0.9, 17.5) |
| 24-months | 5.7 (-2.9, 14.3) | 4 (0.7, 7.4) | 1.6 (-7.5, 10.7) |
